# Cytotoxic effect of different *Uncaria tomentosa* (cat’s claw) extracts, fractions on normal and cancer cells: a systematic review

**DOI:** 10.3389/fphar.2025.1584840

**Published:** 2025-05-14

**Authors:** Adriana A. Lopes, Juliana da Silva Coppede, Pedro de Pádua G. Amatto, Davi Casale Aragon, Suzelei de Castro França, Fabio Carmona, Ana Maria S. Pereira

**Affiliations:** ^1^ Biotechnology Unit, University of Ribeirão Preto (UNAERP), Ribeirão Preto, São Paulo, Brazil; ^2^ Ribeirão Preto Medical School, University of São Paulo, Ribeirão Preto, São Paulo, Brazil; ^3^ Botanical Garden of Medicinal Plants Ordem e Progresso, Jardinopólis, São Paulo, Brazil

**Keywords:** half-maximal inhibitory concentration (IC50), keratinocytes, oxindole alkaloids, proanthocyanidins, Rubiaceae

## Abstract

**Background:**

*Uncaria tomentosa* (cat’s claw) is a medicinal plant with documented immunomodulatory and anti-inflammatory properties. Recent studies suggest potential anticancer effects, but evidence remains fragmented.

**Objective:**

This systematic review aimed to assess the cytotoxic effects of different *U. tomentosa* extracts or fractions on normal and cancer cells, summarizing *in vitro* studies.

**Methods:**

A systematic search was conducted in PubMed, Embase, and Scielo up to January 2025, following PRISMA guidelines. Inclusion criteria comprised *in vitro* studies evaluating *U. tomentosa* extracts or fractions on normal and cancer cells, reporting IC_50_ values or equivalent measures. Data on plant part, extraction method, and chemical composition were collected. Risk of bias was assessed using the modified CAMARADES checklist.

**Results:**

Thirteen studies met the eligibility criteria. *U. tomentosa* extracts exhibited selective cytotoxicity in some cancer cell lines. The most promising findings were observed for crude aqueous bark extracts (72 h incubation) against squamous cell carcinoma and pentacyclic oxindole alkaloid (POA)-rich extracts against prostate cancer and leukemia. In contrast, tetracyclic oxindole alkaloid (TOA)- and proanthocyanidin (PAC)-rich fractions showed limited cytotoxicity. Most extracts were non-toxic to normal cells, except for the crude aqueous bark extract, which exhibited cytotoxicity in keratinocytes.

**Conclusion:**

*U. tomentosa* has potential as a source of selective anticancer agents, particularly through crude aqueous bark and POA-rich extracts. The observed cytotoxic effects vary considerably depending on the extraction method and chemical composition, underscoring the need for standardization in future studies. Further standardized studies and mechanistic investigations are required to validate its therapeutic potential for cancer treatment.

**Systematic Review Registration:**

https://osf.io/hfazq/.

## Highlights

• *Uncaria tomentosa* extracts have selective cytotoxicity against a few cancer cell lines.
• *Uncaria tomentosa* extracts are non-toxic to normal cell lines.
• Pentacyclic oxindole alkaloid (POA)-rich extracts show the highest anticancer potential.
• Tetracyclic oxindole alkaloid and proanthocyanidin fractions exhibit limited cytotoxic effects.
• Most of *U. tomentosa* extracts are safe for normal cells, except for a crude aqueous and POA-rich extracts.

## Introduction

Cancer remains a leading cause of morbidity and mortality worldwide, with an estimated 19.3 million new cases and approximately 10 million deaths reported in 2020 (Sung et al., 2021). Despite advancements in treatment, the global cancer burden continues to rise, highlighting an urgent need for novel, effective, and less toxic therapeutic options. Natural products have historically been a rich source of anticancer agents, and the exploration of medicinal plants offers promising avenues for drug discovery (Newman and Cragg, 2020).

*Uncaria tomentosa* (Willd. ex Schult.) DC. (Rubiaceae), commonly known as “cat’s claw,” is a vine native to the Amazon rainforest and has been traditionally used in South American medicine. This species has high medicinal properties and economic value in the world. In Brazil, this species is distributed in the states of Acre, Amapá, Amazonas, and Pará (Honório et al., 2016), and is disseminated by the Brazilian Ministry of Health to all municipalities through the National Health System (*Sistema Único de Saúde*, SUS) (BRASIL, 2022). The World Health Organization (WHO) described the traditional uses for cat’s claw as being applicable for diverse diseases and illnesses such as arthritis, rheumatism, gastric ulcers, abscesses, asthma, fevers, urinary tract infections, viral infections, wounds and as an emmenagogue (WHO, 2007). The plant is rich in bioactive compounds including pentacyclic (POA), tetracyclic (TOA) oxindole alkaloids, triterpenes, quinic acid esters, polyphenols (phenolic acids and proanthocyanidins), flavonoids, quinones, and glycosides, which have been attributed to its diverse pharmacological properties (Batiha et al., 2020). Notably, extracts from *U. tomentosa* exhibit immunomodulatory, anti-inflammatory, and potential anticancer activities (Araujo et al., 2018; Batiha et al., 2020; Blanck et al., 2022; Rojas-Duran et al., 2012; Urdanibia et al., 2013).

Tetracyclic oxindole alkaloids (TOA) act on the central nervous system. For instance, isorhynchophylline can improve memory problems by increasing the antioxidant levels, while D-galactose exerts an anti-inflammatory effect on brain tissues in mice (Xian et al., 2014). POA affect the immunocellular system (Reinhard, 1999), increasing the rate of phagocytosis by granulocytes (Wagner et al., 1985) and inducing lymphocyte specificity (Wurm et al., 1998).

Tetracyclic oxindole alkaloids (TOA) act mostly on the central nervous system, while POA affect the immunocellular system (Zhang et al., 2015). The POA mitraphylline and isopteropodine are the chemical markers used in the quality control of cat’s claw herbal medicine (USP-United States Pharmacopeia, 2022). Nevertheless, in recent years, the anticancer activity of this plant has been explored (Dreifuss et al., 2013; Kaiser et al., 2013).

A promising anticancer compound should exhibit minimal or no toxicity to normal cells and possess a clearly defined mechanism of action. The most common antitumor mechanism is the induction of apoptosis, a programmed cell death pathway that is often dysregulated in cancer cells. Several natural products are capable of reactivating apoptotic signaling in cancer cells. Oxindole alkaloids are particularly potent in their anticancer activities. They can induce apoptosis, inhibit cell proliferation, and exhibit cytotoxic effects against various cancer cell lines, making them valuable in the search for effective cancer therapies (Khetmalis et al., 2021; Kozielewicz et al., 2014).

Mitraphylline isolated from *U*. *tomentosa* bark showed cytotoxic effect on human Ewing’s sarcoma and breast cancer cell lines (García Giménez et al., 2010). Pteropodine, a POA from *U. tomentosa*, induced apoptosis in T lymphoblastic cells independently of the CD95/Fas death receptor pathway (Bacher et al., 2006). Moreover, both an *n*-butanol-soluble fraction and a hydroalcoholic extract of *U. tomentosa* activated caspase-3, a key executioner enzyme in the apoptotic cascade (De Martino et al., 2006; de Oliveira et al., 2014). Similarly, an ethyl acetate extract of *U. tomentosa* induced apoptosis through caspase activation in HL-60 leukemia cells, potentially engaging either mitochondrial (intrinsic) or receptor-mediated (extrinsic) pathways (Cheng et al., 2007).

Despite a growing body of evidence supporting the anticancer activity of various *U. tomentosa* extracts and isolated compounds, it remains unclear which specific extract types or chemical constituents are most effective against particular cancer cell lineages. Given the pharmacological relevance and chemical diversity of *U. tomentosa*, this systematic review aims to summarize *in vitro* studies investigating its anticancer properties, with a focus on the bioactivity of chemically distinct extracts and fractions derived from different parts of the plant.

## Methods

This report was based on Preferred Reporting Items for Systematic Reviews and Meta-Analyses (PRISMA) Guidelines (Page et al., 2021). A protocol was deposited on the Open Science Framework (Carmona and Pereira, 2025).

### Inclusion and exclusion criteria

This systematic review included only *in vitro* studies on the effects of *U. tomentosa* extracts (crude, fraction, or purified) on normal or cancer cells, and that reported half-maximal inhibitory concentrations (IC_50_) or other values that allow IC_50_ estimation. *In vivo* studies and those on mixtures of medicinal plants or isolated substances were not included. Only studies published in English, Portuguese, or Spanish were included.

### Source of information and search strategy

Only articles published from inception to January 2025, were searched in the following databases: PubMed, Embase, and Scielo. The initial publication date was not limited. The query search used in Embase for this study is presented in Box 1. Similar queries were employed in the other databases. The reference lists of all selected studies were also searched to identify additional primary studies for inclusion.

Box 1 **Search queries used in Embase for the systematic review.**

(“cancer cell/exp OR “cancer cell” OR “cancer cell model” OR “cancer cells” OR “cancerous cell” OR “cancerous cells” OR “cell, cancer” OR “malignancy cell” OR “malignancy cells” OR “malignant cell” OR “malignant cells” OR “malignant tumor cell” OR “malignant tumour cell” OR “oncocyte” OR “oncocytes” OR “cancer cell line/exp OR “cancer cell line” OR “cancer cell lines” OR “cancer cell strain” OR “cancer derived cell line” OR “cancer line” OR “cancerous cell line” OR “malignancy cell line” OR “malignancy-derived cell line” OR “malignant cell line” OR “malignant line” OR “cells/exp OR “cell” OR “cells”) AND (“uncaria tomentosa/exp OR “uncaria tomentosa” OR “cat`s claw”) AND (“ic50/exp OR “cytotoxicity/exp OR “cell toxicity” OR “cytotoxic activity” OR “cytotoxic effect” OR “cytotoxic reaction” OR “cytotoxicity” OR “apoptosis/exp OR “ap-optosis” OR “apo-ptosis” OR “apoptosis” OR “apoptotic cell death” OR “apoptotic cellular death” OR “apoptotic death” OR “apoptotic suicide” OR “apoptotic-like cell death” OR “cell suicide” OR “cellular suicide” OR “programmed cell death” OR “programmed cell death type i OR “suicidal cell death)

### Article selection

Two independent and initially blind reviewers (AAL, JSC, and PPGA) conducted the screening of articles by reading titles and abstracts. The entire process used the Rayyan software (Ouzzani et al., 2016). Disagreements were resolved by consensus among the reviewers (with a fourth reviewer, AMSP, when necessary). A PRISMA flowchart was built using the PRISMA Flow Diagram tool (Haddaway et al., 2022).

### Data collection

Information on the plant parts used, methods of extraction, fractioning, or purification, extraction times, and the different types of cells studied were retrieved, whenever available. IC_50_ values were extracted from individual studies or calculated from MTT results [log (inhibitor) vs response, variable slope with four parameters] using GraphPad Prism 10 (LaJolla, CA, United States). When values were reported in charts, they were estimated by measuring bar heights using ImageJ (Schneider et al., 2012). The authors of the included studies were contacted when necessary (when some data or article was not available). The retrieved data were recorded in a Google Sheets spreadsheet (Alphabet Inc., Mountain View, CA, United States).

### Quality assessment

For the risk of bias, two investigators (AMSP and FC) independently reviewed the selected studies according to a modified CAMARADES checklist (Macleod et al., 2004) and reported the risks of bias in a table.

### Statistical analysis

Heatmaps for normal and cancer cells were plotted using the package *pheatmap* of R 4.3.2 (The R Foundation), and GraphPad Prism 10 (GraphPad, LaJolla, CA, United States), indicating the classes of major compounds of each extract or fraction and the embryonic origin of each cell tested. If more than one IC_50_ was obtained for the same extract and cell lineage, the average value was used.

To categorize the therapeutic potential based on IC_50_, the following arbitrary cutoff points were adopted, loosely inspired by previous studies (Badisa et al., 2009; Coussens and Werb, 2002; Shoemaker, 2006; Zhang et al., 2024):

• IC_50_ ≤ 10 μg/mL–Toxic (red color). Values in this range are typically considered highly potent, indicating strong cytotoxic activity, which is desirable against tumor cells, but not normal cells.
• IC_50_ 10–50 μg/mL–Potentially toxic (orange color). This range is still considered promising for anticancer drugs, especially in resistant tumor cells, but for normal cells it is not considered safe.
• IC_50_ 50–100 μg/mL–Potentially safe (yellow to green color). This range may be acceptable depending on the clinical context, especially if the drug shows high selectivity for tumor cells. However, potential toxicity to normal cells should be carefully monitored.
• IC_50_ ≥ 100 μg/mL–Safe (blue color). These values are considered indicative of low cytotoxicity, which is desirable for normal cells, but ineffective for cancer cells.

The National Cancer Institute (NCI) guidelines traditionally consider compounds with IC_50_ ≤ 10 µM (or µg/mL for crude extracts) as having high cytotoxic potential, especially in the NCI-60 human tumor cell line screen (Shoemaker, 2006). Classification ranges for natural products in breast cancer screening studies, with similar thresholds adapted to herbal extract data were also described (Zhang et al., 2024). In a cytotoxicity framework based on extract potency, IC_50_ values > 100 μg/mL are generally deemed non-toxic, while values < 10 μg/mL are considered highly cytotoxic (Badisa et al., 2009). These thresholds are not absolute and are primarily used *in vitro* as screening indicators for selectivity and potency. Additional *in vivo* studies would be necessary to establish clinical safety.

## Results

**FIGURE 1:**
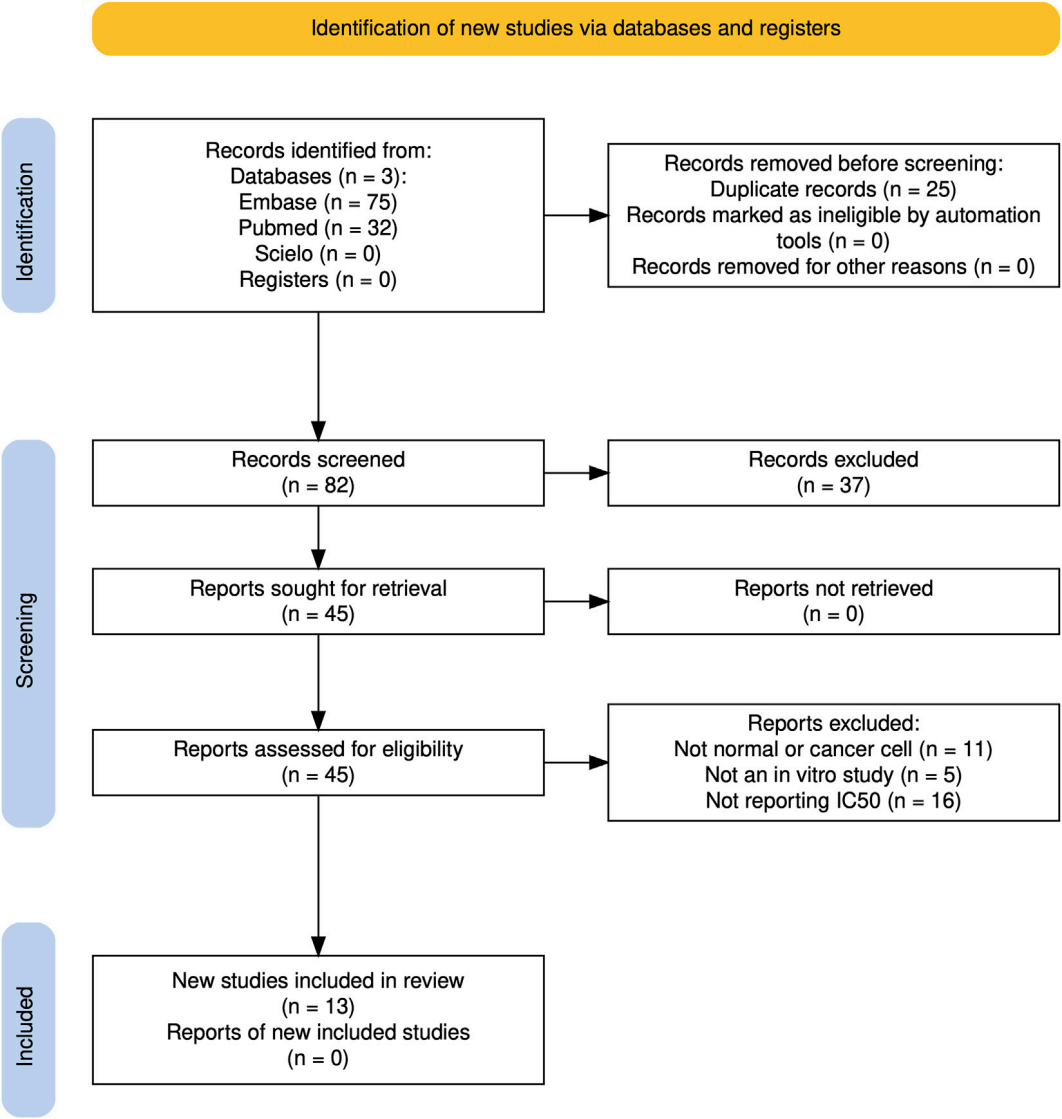
Flowchart of study selection.

**FIGURE 2:**
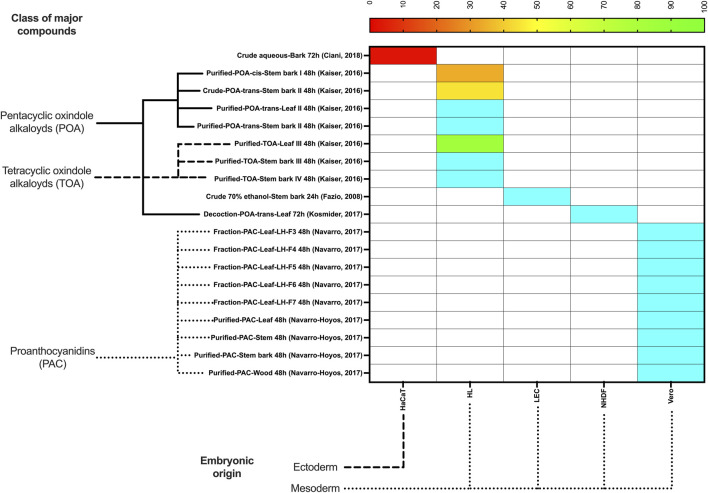
Half maximal inhibitory concentration (IC_50_, in µg/mL) of selected *Uncaria tomentosa* extracts to normal cells. Legend: Values in shades of yellow and green (IC_50_ 50–100 μg/mL) or blue (IC_50_ ≥ 100 μg/mL) are the safest to normal cells. Values in orange are potentially toxic (IC_50_ 10–50 μg/mL), while those in red (IC_50_ ≤ 10 μg/mL) are the most toxic to normal cells. POA, pentacyclic oxindole alkaloids; TOA, tetracyclic oxindole alkaloids; PAC, proanthocyanidins; QAPF, quinovic acid glycosides purified fraction.

Only 13 studies met the inclusion criteria (Figure 1) and are summarized in Table 1. Regarding toxicity to normal cells, a crude ethanolic stem bark extract, PAC-rich fractions or purified extracts, and a POA-trans-rich decoction were non-toxic (IC_50_ > 100 μg/mL) to Vero, NHDF, and LEC cells. The purified TOA-rich extract was potentially non-toxic (IC_50_ 50–100 μg/mL), while crude POA-trans-rich extract bark and the purified POA-cis-rich extract bark were potentially toxic (IC_50_ 10–50 μg/mL) to HL cells. The most worrisome case was the crude aqueous extract (72 h) of the bark, which was toxic (IC_50_ < 10 μg/mL) to HaCaT cells (Figure 2).

**TABLE 1:** Summary characteristics of the included studies.

Plant part, extract preparation, and chemical composition	Model/experimental methods	Main results	Authors’ conclusion	References
Part not specified. *U. tomentosa* powder (70 g) (cat’s claw) was extracted three times with 250 mL each of hexane, ethyl acetate, n-butanol, and methanol. CC-H (n-hexane extracts), CC-EA (ethyl acetate extracts), CCB (n-butanol extracts) and CC-M (methanol extracts). Chemical composition: no data	Cytotoxicity assay using HL-60 (human leukemia cell lines). Dose: *U. tomentosa* extracts CC-H, CC-EA, CC-B and CC-M at 20, 40, 60, 80, 100, 200 μg/mL. Duration: assays were maintained for 12 h	Hexane and ethyl acetate extracts were effective in the concentration range of 13–13.5 μg/mL. The ethyl acetate extract showed a time- and dose-dependent apopitotic effect at a concentration of 100 μg/mL above 6 h	The CC-EA (ethyl acetate extracts) was able of inducing apoptosis in HL-60	Cheng et al. (2007)
Root bark. The aqueous extract (UT-ext) was prepared by 15 g of dried bark were shredded and boiled for 30 min in 500 mL of bi-distilled H_2_O, then cooled at room temperature. Chemical composition of the UT-ext: 1.28 mg/mL of proteins as determined by Bradford assay, 3.08 mg/mL phenols and 4.05 mg/mL carboidrates (2.08 pentoses and 1.97 hexoses). Furthermore, the chemical investigation showed the presence of hydrophilic low-medium molecular weight compounds responsible for anticancer potential	Cell viability assay - MTT colorimetric assay using HaCaT, (keratinocytes from adult skin), A431 (human epidermoid carcinoma cells) and SCC011, SCC013 and SCC022 (head and neck cancer cells) lines. Dose: UT-ext was used from 0.3 to 10 mg/mL. Duration: assays were maintained for 24 h, 48 h and 72 h	UT extract was clearly cytotoxic and exhibited a dose-dependent effect in all tested cell lines. UT-ext at concentration of 0.3–1.5 mg/mL (for 24 h) was effective in inducing cell death of HaCat keratinocytes and head and neck cancer (SCC011, SCC013 and SCC022)	SCCs cells are more susceptible to UT-ext treatment	Ciani et al. (2018)
Stem bark. The crude extract was prepared by 4 days-maceration with ethanol:water solution (40%, v:v) in a stem bark:solvent ratio of 1:10 (w:v). Chemical composition: presence of glycosylated quinovic acids	Cell viability assay - MTT colorimetric assay using T24 and RT4 (human bladder cancer) cells. Dose: *U. tomentosa* extract at 5, 10, 25, 50, 100 and 150 μg/mL. Duration: assays were maintained for 48 h	The antiproliferative effect of the ethanol-water extract was demonstrated against the T24 and RT4 (bladder cancer) lines at a concentration of 150 μg/mL after 48 h of treatment	The authors point out that the presence of glycosylated quinovic acids is responsible for the activity	Dietrich et al. (2014)
Bark. Aliquots were ground and macerated in a 70% ethanol in water solution for 21 days in the dark at room temperature. Chemical composition: no data	Cytotoxic assay was performed using B16/BL6 (murine melanoma), K1735 (amelanotic murine melanoma), HT29 (human colon carcinoma), A549 (human lung carcinoma), WEHI 164 (mouse fibrosarcoma) and LEC (mouse liver endothelial) cells line. Dose: *U. tomentosa* extract at 1, 10, 100 and 300 μg/mL. Duration: assays were maintained for 24 h	Inhibitory effect of hydroalcoholic extract was verified on 24 h of cell growth only at the higher concentrations of 100 and 300 μg/mL, less than 50% in all cells tested	The hydroalcoholic extract (70%) evaluated on different cells of melanoma (B16, K1735), colon carcinoma (Ht29), lung carcinoma (A549), fibrosarcoma (WEHI164) and liver endothelium (LEC), showed an inhibition of less than 50%	Fazio et al. (2008)
Bark. 500 g of *Uncaria tomentosa* dried inner bark were treated with ammonium hydroxide and dichloromethane. After filtration, the obtained solution was concentrated *in vacuo* to afford a residue, which was dissolved in a hydrochloric acid solution (3%). Ammonium hydroxide and dichloromethane were added again. After concentration *in vacuo*, the purified alkaloid fraction was obtained as a brown residue and the yield was 0.15%. Chemical composition: 87.3% mitraphylline in dry extract	Cell proliferation assay - using human Ewingʼs sarcoma MHH-ES-1 and breast cancer MT-3 cell lines. Doses: 5, 10, 20, 30 and 40 μM of mitraphylline. Duration: assays were maintained for 30 h	The IC_50_ ± SE values were 17.15 ± 0.82 μM for MHH-ES-1 and 11.80 ± 1.03 μM for MT-3 for 30 h	Mitraphylline might be a new promising agent in the treatment of both human sarcoma and breast cancer	García Giménez et al. (2010)
Bark. One gram of the bark was extracted in 10 mL of water for 8 h at 37°C (B/W_37_, aqueous extract of the bark). An alkaloid-rich bark extract was prepared by 10 g of bark was extracted with 50 mL of water (6 h, 37°C) followed of addition of 50 mL of dichloromethane and organic layer was evaporated (B/S_rt_). Chemical composition: alkaloid-enriched extract	WST1-cell viability assay using SW480 (human colorectal cancer), HeLa (human cervical carcinoma) and 293T (human embryonic kidney epithelial) cell lines. Dose: *U. tomentosa* extract at final concentrations of 1 μg/mL to 300 μg/mL). Duration: assays were maintained for 0, 2, 5, or 23 h	The B/S_rt_ extract, rich in alkaloids, was effective for HeLa, HCT616 and SW480 cells. The IC_50_ for B/Srt after 24 h was lower than that of B/W_37_, indicating a stronger effect on cell proliferation	The alkaloid-enriched extract (B/S_rt_) was found to be more effective than the aqueous extract (B/W_37_)	Gurrola-Díaz et al. (2011)
Stem bark and leaf. The extracts were prepared by 10 g of powdered samples (stem bark or leaf) into hydroethanolic solution 63% (v/v) at a plant: solvent ratio of 0.5:10 (w/v) by 2-h dynamic maceration in a magnetic stirrer at 300 rpm. Purified fractions (SI, SII, SIII, SIV, LII, LIII) and crude extract (CESII). SI: chemotype I (pentacyclic oxindole alkaloids (POA) with cis D/E ring junction). SII, LII and CESII: chemotype II (POA with trans D/E ring junction). SIII, SIV and LIII: chemotype III (tetracyclic oxindole alkaloids (TOA)). Chemical composition: alkaloid content ranged from 19.66% to 87.89% (w/w) and seemed unrelated to any of the chemotypes analyzed	Cell viability assay - MTT colorimetric assay using malignant cell lines T24 (human bladder cancer cell line) and U-251-MG (human glioblastoma cell line). Dose: concentrations ranging from 18.7 to 935.4 μM and 1.2–59.5 μM, respectively were employed for the cytotoxicity evaluation of the purified fractions and crude extract. Duration: assays were maintained for 48 h	Standardized hydroalcoholic extract of chemotypes I, II and III showed similar cytotoxicity against malignant cells, and chemotypes I and III showed greater cytotoxicity against normal cells. Chemotype 2 was more selective against malignant cell lines	Chemotypes I, II and II showed different selectivity against human malignant cells	Kaiser et al. (2016)
Leaves. *U. tomentosa* extract was prepared by 4 g of leaves were extracted with warm water (200 mL, boiling point temperature) for 20 min and 1.16 g of dry powder was obtained. Chemical composition: extract contains POA (13% of dry extract mass) and is free of TOA.	Cell viability assay - MTT colorimetric assay using HepG2 (human hepatoma) and NHDF (fibrolast) cells. Dose: *U. tomentosa* extract at 72.5–145 μg/mL. Duration: assays were maintained for 72 h	Extract standardized extract (aqueous leaf), containing POA and free of TOA, showed cytotoxicity against HepG2 (10%–15% decrease in cell viability) but not showed cytotoxicity in normal cells (NHDF), there is a modulatory effect between tumor and normal cells	*U. tomentosa* extract was not cytotoxic for NHDF cells but demonstrated cytotoxic effect against HepG2 cells	Kośmider et al. (2017)
Leaves. The dried material (0.05 g/mL) was first extracted with a mixture of methyl ter-butyl ether (MTBE) and methanol (MeOH) 90:10 (v/v) during 30 min in ultrasound to obtain a non-polar extract. Residual material was extracted with MeOH during 30 min in ultrasound and MeOH was evaporated, to deliver a polyphenolic-rich extract. Methanolic extract (polyphenolic extract) was loaded in Dovex to get *U. tomentosa* fractions. Chemical composition: Protoanthocyanidins-rich fraction - procyanidins (46%) and propelargonidins (37%)	Cell viability assay - MTT colorimetric assay using SW 620 (human colorectal colon adenocarcinoma cell line), AGS (human gastric adenocarcinoma), and Vero cells (monkey normal epithelial kidney cells). Dose: 15–500 μg/mL final concentration (DMSO 0.1% v/v). Duration: assays were maintained for 48 h	Protoanthocyanidins-rich fraction from leaf extract showed cytotoxic activity for gastric adenocarcinoma AGS and SW620 colon rectal at a concentration of 15–500 μg/mL	These finds showed the potential health effects of *U. tomentosa* proanthocyanidin extracts on gut-related diseases, such as colon cancer	Navarro et al. (2017)
Leaves, stems, bark and wood. *U. tomentosa* extract was prepared from dried material in a mixture (0.05 mg/mL) of methyl ter-butyl ether (MTBE) and methanol (MeOH) 90:10 (v/v) during 30 min in ultrasound (to remove non-polar compounds). MTBE-MeOH extract was washed with MeOH and evaporated (to remove the polyphenolic-rich compounds). Then, residual material was extracted with MeOH during 30 min in ultrasound and concentrated. These methanol extracts and the MeOH washings were combined and washings with hexane, MTBE and chloroform. Chemical composition: all extracts contain pure procyanidins or mixed proanthocyanidins with different (epi)catechin/(epi)afzelechin units	Cell viability assay - MTT colorimetric assay using AGS (human gastric adenocarcinoma), SW620 (human colorectal adenocarcinoma) and Vero (monkey normal epithelial kidney) cells. Dose: *U. tomentosa* extract at 111–500 μg/mL. Duration: assays were maintained for 48 h	Methanolic extracts rich in polyphenols (protoanthocyanins) showed strong action against adenocarcinoma cell lines (AGS; SW620); AGS cells: IC_50_ for leaves and bark extracts: 116–195 and 111–220 μg/mL; SW620 cells: IC_50_ for leaves and bark extracts: 118–160 and 111–142 μg/mL. The growth in VERO cells (normal cells) was not affected at concentrations above 500 μg/mL	The potential value of *U. tomentosa* -especially leaves and bark- polyphenolic extracts	Navarro-Hoyos et al. (2017)
Bark. One gram of the bark was extracted in 10 mL of water, 25%, 50%, and 96% ethanol for 8 h at 37°C (B/W_37_, B/50E_37_ and B/96E_37_, respectively). For water and 96% ethanol extractions were also performed at boiling temperature of these solvents for 8 h (preparations B/Wb, and B/Eb). B/S_rt_ - bark alkaloid-rich preparation: Ten grams of the *U. tomentosa* bark were extracted with 50 mL of water (6 h, 37°C). Next, the sample was centrifuged at 35,000 rpm. To the water supernatant 50 mL of dichloromethane was added and the mixture was intensively shaken. The organic layer was evaporated and dissolved in 1.5 mL of 96% of ethanol (stock solution). Chemical composition: Ethanolic extract showed the presence of alkaloids pteropodine/isomitraphylline	Antiproliferative assays was performed using HT-29 (colon adenocarcinoma), SW707 (human colorectal adenocarcinomas), KB (human cervical carcinoma), MCF7 (human breast carcinoma), A549 (human non-small cell lung carcinoma), OAW-42 (human ovarian cystoadenocarcinoma), HL60 (human acute promyelocytic leukemia), LLC (LL/2, mouse Lewis lung carcinoma) and B16 (mouse melanoma). Dose: B/W37, B/Wb, B/50E37, B/Eb, B/96E37 and B/SRT extracts at 1,000 and 23.57 μg/mL. Duration: assays were maintained for 72 h	The B/S_RT_ extract showed the highest growth inhibition for human cervical carcinoma (KB) [IC_50_ = 25.57 μg/mL] and the B/96E_37_ extract showed the best inhibition for mouse Lewis lung carcinoma (LL/2) [IC_50_ = 25.06 μg/mL], thereby ethanol preparations have increased action as compared to aqueous ones	The bark extract prepared with different solvents and in different temperatures (37°C and boiling conditions) produces dissimilar results in all cancer lines evaluated, and this is also true for the alkaloid content which is variable depending on the preparations; the cell dies depending on concentration of alkaloid content	Pilarski et al. (2010)
Bark. Ten grams of the bark were powdered and extracted with 50 mL of water (6 h, 37°C). Then, 50 mL of dichloromethane was added, and the mixture was vigorously shaken. The organic layer was evaporated (150 mg) and dissolved in 1.5 mL of 96% ethanol (BSRT). Chemical composition: 50% of POA on BSRT with predominance of pteropodine, speciophylline and isopteropodine	Cell viability assay - MTT colorimetric assay using HL-60 (human leukemia cell lines). Dose: BSRT extract at 50 ng/mL or 50 μg/mL. Duration: assays were maintained for 24 and 48 h	The BSRT extract showed anti-leukemic activity against the HL-60 cells with IC_50_ = 60 μg/mL and this effect was two times higher in comparison to the control	The POA are main active compounds present in BSRT extract, so it was supposed that they were responsible for this effect	Pilarski et al. (2013)
Stem bark. UT extract was prepared by dispersing 1 g of dried UT extract into 50 mL of each organic solvent: ethyl acetate (EA) and acetone (AC). Then, the proportion of solvent mixture was prepared: 3 mL of ethyl acetate (EA) and 2 mL of acetone (AC), with or without UT extract. Chemical composition of the solvent mixture: 20 mg/mL of UT extract for each solvent (1.4 mg of total alkaloids POA in the 5 mL of the solvents mixture)	Cell viability assay - MTT colorimetric assay using two prostate cancer cell-lines, DU145 (androgen-insensitive human PC cell) and the LNCaP (androgen-sensitive human PC cell) lines. Dose: UT extract was used at five different concentrations of alkaloids (40–200 mg/mL), which were diluted in culture medium at a ratio of 1:10 and resulted in concentrations ranging from 4.0 to 20.0 mg/mL. Duration: assays were maintained for 24 h, 48 h and 72 h	UT extract was able to reduce cell viability of the PC cell lines LNCaP and DU145 in a time and concentration-dependent manner. The DU145 cells were more sensitive at higher concentrations than LNCaP cells. Higher activity against DU145 cells: IC_50_ = 15.7 mg/mL of total alkaloids, at 72 h	Results suggest that this UT extract has potential as anti-cancer alternative	Ribeiro et al. (2020)

Legend: A-549, Human lung carcinoma cell line; AGS, human gastric adenocarcinoma cell line; B/50E37, Ethanolic extract (50%) of *Uncaria tomentosa* bark prepared at 37°C; B/96E37, Ethanolic extract (96%) of *Uncaria tomentosa* bark prepared at 37°C; B/Eb, Ethanolic extract of *Uncaria tomentosa* bark prepared at boiling temperature; B/Srt, Alkaloid-rich bark extract of *Uncaria tomentosa*; B/W37, Aqueous extract of *Uncaria tomentosa* bark prepared at 37°C; B/Wb, Aqueous extract of *Uncaria tomentosa* bark prepared at boiling temperature; B16, Mouse melanoma cell line; CC-B, *uncaria tomentosa* butanolic extract; CC-EA, *uncaria tomentosa* ethyl acetate extract; CC-H, *uncaria tomentosa* hexane extract; CC-M, *uncaria tomentosa* methanol extract; CDKs, Cyclin-dependent kinases; CESII, Crude extract of *Uncaria tomentosa* containing chemotype II; DU145, Androgen-insensitive human prostate cancer cell line; EA, ethyl acetate; HeLa, Human cervical carcinoma cell line; HL-60, Human acute promyelocytic leukemia cell line; KB, human cervical carcinoma cell line; LNCaP, Androgen-sensitive human prostate cancer cell line; LL/2, mouse lewis lung carcinoma cell line; MHH-ES-1, Human Ewing’s sarcoma cell line; MT-3, human breast cancer cell line; NHDF, normal human dermal fibroblast cells; PAC, proanthocyanidins; POA, pentacyclic oxindole alkaloids; QAPF, quinovic acid glycosides purified fraction; SCC011, Human squamous cell carcinoma cell line; SCC013, Human squamous cell carcinoma cell line; SCC022, Human squamous cell carcinoma cell line; SI, Chemotype I extract (pentacyclic oxindole alkaloids with cis D/E ring junction); SII, Chemotype II, extract (pentacyclic oxindole alkaloids with trans D/E ring junction); SIII, Chemotype III, extract (tetracyclic oxindole alkaloids); SIV, Chemotype III, extract (tetracyclic oxindole alkaloids); SW480, Human colorectal adenocarcinoma cell line; SW620, Human colorectal adenocarcinoma cell line; T24, Human bladder cancer cell line; TOA, tetracyclic oxindole alkaloids; U-251-MG, human glioblastoma cell line; UT-ext, *Uncaria tomentosa* extract; VEGF, vascular ndothelial growth factor; Vero, Monkey kidney epithelial cell line; WEHI 164, Mouse fibrosarcoma cell line.

**FIGURE 3:**
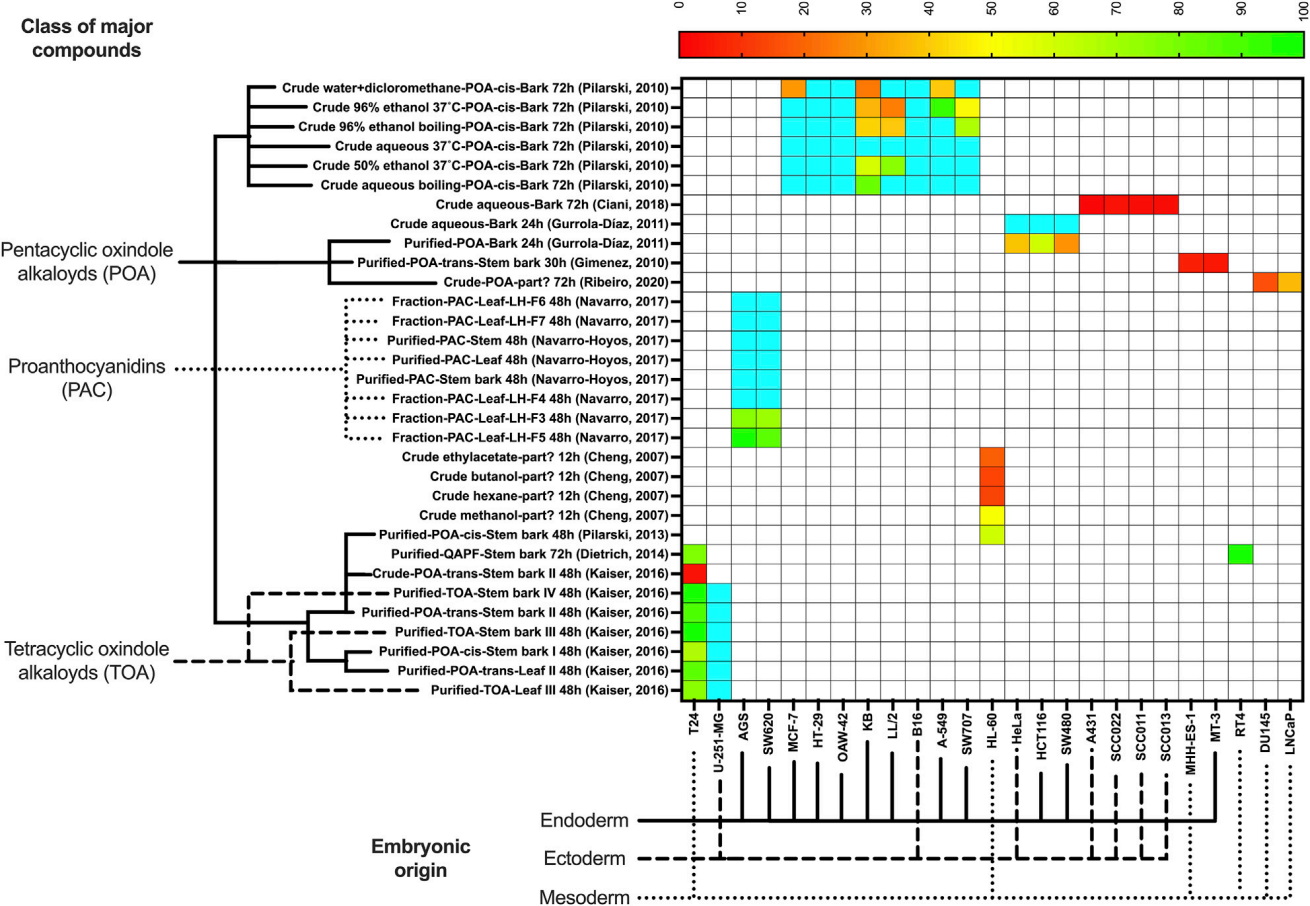
Half maximal inhibitory concentration (IC_50_, in µg/mL) of selected *Uncaria tomentosa* extracts to cancer cells. Legend: Values in shades of red (IC_50_ ≤ 10 μg/mL) are the most promising, while those in shades of orange (IC_50_ 10–50 μg/mL) are potentially promising. Values in yellow to green (IC_50_ 50–100 μg/mL) or blue (IC_50_ ≥ 100 μg/mL) are not promising to treat cancer. POA, pentacyclic oxindole alkaloids; TOA, tetracyclic oxindole alkaloids; PAC, proanthocyanidins; QAPF, quinovic acid glycosides purified fraction.

Regarding toxicity to cancer cells, most extracts were non-toxic or only potentially toxic. Some preparations (extracts or fractions) showed clear toxicity in cancer cells, such as the crude aqueous bark extract (72 h) mostly for ectoderm-origin cancer cells (A431, SCC011, SCC013, and SCC022), the purified POA-trans-rich stem bark extract to MT-3 and MHH-ES-1cells, and the crude POA-trans-rich stem bark extract to T24 cells. Other promising potentially toxic extract were the crude hexane, butanolic, and ethylacetate extracts against HL-60 cells, the water + dicloromethane extract against A-549, KB, and MCF-7 cells, the crude POA-rich extract (72 h) against DU145 and LNCaP cells, the POA-cis-rich bark extract against HeLa and SW480 cells, and the POA-cis-rich crude 96% ethanolic extracts against KB and LL/2 cells. All PAC-rich fractions or purified extracts, from either leaf or bark, were nontoxic to cancer cells AGS and SW620 (Figure 3).

The risk of bias assessment (Table 2) revealed several methodological limitations across the included studies. Although all articles were published in peer-reviewed journals and most provided adequate control of temperature and reported outcome data, crucial elements such as blinding of outcome assessment and randomization were rarely addressed, being marked as “unclear” in over 90% of the studies. Additionally, nearly half of the studies did not include a non-cancer cell lineage control, which limits interpretation of selectivity.

**TABLE 2:** Risk of bias among the included studies.

Criteria										
Publication in a peer-reviewed journal	Control of temperature	Non-cancer cell lineage control	Apropriate number of replicates	Blinding of the outcome assessor	Random outcome assessment	Incomplete outcome data	Selective outcome reporting	Other sources of bias	Possible conflict of interest	References
Low	Low	High	Unclear	Unclear	Unclear	Low	Low	Low	Unclear	Cheng et al. (2007)
Low	Low	Low	Low	Unclear	Unclear	Low	Low	Low	High	Ciani et al. (2018)
Low	Low	High	Unclear	Unclear	Unclear	Low	Low	Low	Low	Dietrich et al. (2014)
Low	High	Low	Low	Unclear	Unclear	Low	Low	Low	Unclear	Fazio et al. (2008)
Low	Low	High	Low	Unclear	Unclear	Low	Low	Low	Low	García Giménez et al. (2010)
Low	Low	Low	Unclear	Unclear	Unclear	Low	Low	Low	Low	Gurrola-Díaz et al. (2011)
Low	Low	Low	Low	Low	Unclear	Low	Low	Low	Low	Kaiser et al. (2016)
Low	Low	Low	Low	Unclear	Unclear	Low	Low	Low	Low	Kośmider et al. (2017)
Low	Low	Low	Low	Unclear	Unclear	Low	Low	Low	Low	Navarro et al. (2017)
Low	Low	Low	Low	Unclear	Unclear	Low	Low	Low	Low	Navarro-Hoyos et al. (2017)
Low	Low	High	Low	Unclear	Unclear	Low	Low	Low	Unclear	Pilarski et al. (2010)
Low	Low	High	Low	Unclear	Unclear	Low	Low	Low	High	Pilarski et al. (2013)
Low	Low	High	Low	Unclear	Unclear	Low	Low	Low	Unclear	Ribeiro et al. (2020)
Summary										Risk
100%	92%	54%	77%	8%	0%	100%	100%	100%	54%	Low
0%	0%	0%	23%	92%	100%	0%	0%	0%	31%	Unclear
0%	8%	46%	0%	0%	0%	0%	0%	0%	15%	High

## Discussion

In this study, we demonstrated that POA-rich *U. tomentosa* extracts exhibit significant cytotoxic activity against various cancer cell lines, including epidermoid carcinoma, squamous cell carcinoma, breast cancer, Ewing’s sarcoma, and bladder transitional cell carcinoma (Batiha et al., 2020; Kaiser et al., 2016). Over 30% (10/32) of the tested extracts were toxic to cancer cells. In contrast, TOA- and PAC-rich extracts did not show promising cytotoxic effects. Importantly, most *U. tomentosa* extracts were non-toxic to normal cells, except some POA-rich extracts and the crude aqueous bark extract studied by Ciani et al. (2018). Nevertheless, the extracts’ overall low toxicity reinforces their potential as selective anticancer agents; however, further studies are necessary to confirm this finding across a broader range of cell types.

Our analysis also revealed that the cytotoxic effects of *U. tomentosa* extracts depend on multiple factors, including the plant part used, the extraction method (e.g., solvent, time, purification), and the specific chemotype (Kaiser et al., 2016). Chemotype I (POA *cis*-D/E ring junction) and chemotype III (TOA) displayed greater cytotoxicity to normal cells, whereas chemotype II (POA *trans*-D/E ring junction) was not toxic to normal cells. Notably, standardized POA-rich extracts (4.5% POA) demonstrated cytotoxic activity to prostate cancer cells (IC_50_ = 15.7 μg/mL) (Ribeiro et al., 2020), and ethyl acetate extracts showed dose- and time-dependent apoptotic effects (Cheng et al., 2007), emphasizing the role of extraction conditions in therapeutic efficacy. Conversely, PAC-rich fractions were nontoxic to normal or cancer cells (Navarro-Hoyos et al., 2017).

Alkaloids exhibit a wide range of biological activities relevant to cancer therapy, such as interfering with the cell cycle [vinblastine and vincristine, from *Catharanthus roseus* (L.) G.Don (*Apocynaceae*)], inducing apoptosis [berberine, from *Berberis vulgaris* L. (*Berberidaceae*)], and inhibiting angiogenesis [camptothecin, from *Camptotheca acuminata* Decne. (*Nyssaceae*)] (Newman and Cragg, 2020). Additionally, oxindole alkaloids can generate reactive oxygen species (ROS), which can lead to oxidative stress and subsequent cell death (Cheng et al., 2007). This property is particularly beneficial as it selectively targets malignant cells while sparing normal cells, reducing side effects commonly associated with chemotherapy. This multifactorial action enhances their anticancer efficacy and helps overcome drug resistance (Batiha et al., 2020). The oxindole nucleus is a fundamental component of numerous anticancer pharmaceutical agents, both those that are currently available and those that are currently under investigation. The majority of oxindole nucleus-containing compounds exhibited anticancer activity upon substituting at the carbon-3 position with the formation of a spiro ring. Moreover, the methoxy group at the 5th position was found to be partially critical for upregulation of tumor suppressor proteins (Khetmalis et al., 2021).

Compounds such as mitraphylline and other POAs have demonstrated significant cytotoxicity against a range of cancer cell lines, including breast cancer, Ewing’s sarcoma, and glioblastoma, with IC_50_ values lower than those of standard chemotherapeutic agents (García Giménez et al., 2010). The anticancer properties of POAs compounds have been demonstrated through a variety of methods. These include inhibition of cell growth, blocking of the cell cycle, and induction of cell apoptosis, among others (Wang et al., 2025). Further exploration into their pharmacological profiles and mechanisms of action is warranted to fully understand their potential in cancer therapy. PACs may exert their anticancer effects via two pathways: the caspase-dependent and the caspase-independent pathways.

Proanthocyanidins (PACs), a class of polyphenolic compounds found abundantly in various plants, have emerged as promising candidates for cancer therapy due to their antioxidant, anti-inflammatory, and antiproliferative properties (Navarro et al., 2017). They can exert anticancer effects through multiple mechanisms, including inducing apoptosis by modulating caspases, Bax, and Bcl-2, inhibiting angiogenesis via VEGF downregulation, and interfering with cell proliferation by modulating cyclins and CDKs (Navarro-Hoyos et al., 2017). PACs have previously demonstrated efficacy against breast, prostate, colorectal, and lung cancers. One of the most promising aspects of PACs is their ability to enhance existing cancer treatments. PACs have the potential to treat resistant and malignant tumors, so researchers investigated PAC activity at the molecular level. These studies found that PACs produce reactive oxygen species to stimulate apoptosis pathways (Albogami, 2020). PACs can sensitize cancer cells to chemotherapy and radiation therapy, inhibit DNA repair pathways, and mitigate chemotherapy-induced oxidative stress and inflammation (Navarro et al., 2017). This dual action makes PACs attractive for combination therapy. Furthermore, PACs are generally well-tolerated and exhibit low toxicity, even at high doses (Navarro-Hoyos et al., 2017). Our findings align with previous studies showing that different *U. tomentosa* extracts selectively target malignant cells while sparing normal cells (Batiha et al., 2020; Kaiser et al., 2016). Standardized hydroethanolic and aqueous extracts enriched in POAs have consistently exhibited cytotoxicity across various cancer types, including hepatoma, adenocarcinoma, and leukemia. However, some studies reported an absence of activity against specific cancer lines, such as MDA-MB-231 breast cancer cells and CHO hamster ovary cells (Kośmider et al., 2017), indicating that the efficacy of *U. tomentosa* may not be universally applicable across all malignancies. Interestingly, while oxindole alkaloids have traditionally been considered the main bioactive compounds, some non-alkaloid fractions also exhibited anticancer activity. Glycosylated quinovic acids contributed to cytotoxicity in bladder cancer cells (Dietrich et al., 2014), and alkaloid-poor fractions inhibited medullary thyroid and breast cancer cell proliferation, though without pro-apoptotic effects (Rinner et al., 2009).

Despite the promising anticancer properties of alkaloids and PACs, several challenges must be addressed in drug development (Urdanibia et al., 2013). Firstly, the cytotoxicity of alkaloids varies significantly depending on their chemical structure and functional groups, influencing their interaction with cellular targets (Kaiser et al., 2016). Secondly, alkaloids and polyphenols, including PACs, often exhibit low water solubility, limiting their formulation for intravenous administration (Newman and Cragg, 2020). Thirdly, cancer cells may develop resistance via efflux pumps (e.g., P-glycoprotein), reducing drug intracellular concentration and effectiveness (Gurrola-Díaz et al., 2011). Fourthly, the biological activity of PACs is influenced by polymerization degree and monomer types, leading to efficacy and safety differences. Developing standardized extracts with consistent composition and potency is crucial for clinical applications (Navarro-Hoyos et al., 2017). Lastly, the high risk of different types of bias identified in the studies may affect the internal validity and reduce the confidence in the pooled conclusions. While the general trend indicates that *U. tomentosa* extracts show selective cytotoxicity with low toxicity to normal cells, the lack of methodological transparency in many studies calls for cautious interpretation and highlights the need for more rigorously designed experiments in future research.

Future research should focus on optimizing formulations and conducting well-designed clinical trials to confirm safety, efficacy, and dosing. The transition from preclinical research to clinical trials requires well-structured studies assessing PACs and alkaloids in cancer patients. Collaborations between researchers, clinicians, and industry stakeholders will be crucial in advancing *U. tomentosa*-derived compounds as viable anticancer agents.

## Conclusion

In conclusion, *U. tomentosa* extracts are promising to treat some types of cancer cells, including epidermoid carcinoma, squamous cell carcinoma, breast cancer, Ewing’s sarcoma, and bladder transitional cell carcinoma. The observed toxicity can be attributed to the presence of POAs and PACs, which promote apoptosis and enhance efficacy against tumors. Moreover, these extracts from *U. tomentosa* are generally safe to normal cells, except POA-rich extracts, which are potentially toxic to these cells.

## Data Availability

The original contributions presented in the study are included in the article/supplementary material, further inquiries can be directed to the corresponding author.
